# Supplementary material for: Dopant-Free Triazatruxene-Based Hole Transporting Materials with Three Different End-Capped Acceptor Units for Perovskite Solar Cells
Source: Nanomaterials (Basel). 2020 May 13;10(5):936. doi: 10.3390/nano10050936 (PMC7279385; doi:10.3390/nano10050936)
Supplement: Supplementary file 1 [file nanomaterials-10-00936-s001.pdf]

Supplementary Materials

Dopant-Free Triazatruxene-Based Hole Transporting Materials with Three Different End-Capped Acceptor Units for Perovskite Solar Cells

Da Rim Kil [1,2,†], Chunyuan Lu [1,2,†], Jung-Min Ji [1,2,†], Chul Hoon Kim [2] and Hwan Kyu Kim [1,2,*]

1 Global GET-Future Lab. Korea University, 2511 Sejong-ro, Sejong 339-700, Korea; ekfla6024@korea.ac.kr (D.R.K.); lcy99168@gmail.com (C.L.); manbbong@korea.ac.kr (J.-M.J.)

2 Department of Advanced Materials Chemistry, Korea University, 2511 Sejong-ro, Sejong 339-700, Korea; chulhoon@korea.ac.kr

† These authors contributed equally to this work.

* Correspondence: hkk777@korea.ac.kr

Characterization. The ^{1}H NMR spectroscopy study was conducted on a Varian Mercury 300 spectrometer (300MHz) using tetramethylsilane (TMS; δ = 0 ppm) as an internal standard. The ^{13}C NMR spectroscopy study was conducted on a Bruker Biospin Gmbh AVAVCEⅡ900 spectrometer using tetramethylsilane (TMS; δ = 0 ppm) as the internal standard. MALDI-TOF mass spectra were recorded on a Bruker Ultra flextreme and MALDI TOF-TOF 5800 system (AB SCIEX, USA). UV/Vis absorption spectra were obtained in chloroform on a Shimadzu UV-2401PC spectrophotometer. Photoluminescence spectra were analyzed with a Fluorolog FL-3-22 fluorimeter from Horiba-Jobin-Yvon Ltd., which was equipped with a 450 W Xe lamp and two analyzing monochromators. Cyclic voltammetry (CV) was carried out with a Versa STAT3 (AMETEK). The three-electrode cell system used comprised of a glassy carbon electrode as the working electrode, and a platinum wire and Ag/$AgNO_3$ as a counter and reference electrode, respectively. The potential values were measured relative to an internal ferrocenium/ferrocene reference (Fc+ /Fc). Freshly distilled, N_2- purged THF was used as the solvent with 0.1 M tetrabutylammonium tetrafluoroborate as the supporting electrolyte. Thermal properties of developed HTMs were analyzed using Sinco DSC N-650 and TGA N-1000. The morphologies of the perovskite films and each HTM spin-coated on top of the perovskite films were examined by field-emission scanning electron microscopy (FESEM, Hitachi S4300). The top view morphology and surface roughness of the naked perovskite films, and various HTMs spin-coated on top of the perovskite films, were measured by AFM (XE-7 Park Systems).

Hole mobility measurements. The hole mobility of each HTM was determined using the space-charge-limited current (SCLC) method, which can be described using the following equation:[1]

$$J = \frac{8}{9}\mu\varepsilon_0\varepsilon_r\frac{V^2}{d_3}$$

where J is the current density, μ is the hole mobility, ε_0 is the vacuum permittivity (8.85 × 10^{-12} F/m), ε_r is the dielectric constant of the material (normally taken to approach 3 for organic semiconductors), V is the applied bias, and d is the film thickness measured through the cross-section using field-emission scanning electron microscopy. The hole only device structure used in this work was FTO/PEDOT:PSS/HTM/Au.

Solar cell performance measurements *J-V* curves were performed using a Keithley model 2400 source measurement unit. A 300W Xenon lamp (Spectra-Physics) was used as the light source, and the light intensity was adjusted by using an NREL-calibrated Silicon solar cell equipped with a KG-5 filter for

approximating AM1.5 G 1 sunlight intensity. The magnitude of the alternate signal was10 mV. The incident photon-to-current efficiency (IPCE) spectra was measured as a function of wavelength from 300 to 900 nm using a specially designed IPCE system (PV Measurements, Inc.).

Fabrication of perovskite solar cells Fluorine-doped tin oxide-coated (FTO) glass was chemically etched with zinc powder and dilute 4M HCl solution, and cleaned by sonication in deionized water, acetone, and ethanol. A 20 ~ 30 nm TiO2 compact layer was deposited on the cleaned FTO glass by spray pyrolysis at 450℃ from a precursor solution of 0.5 mL titanium diisopropoxide bis(acetylacetonate) solution in 19.5mL anhydrous ethanol. Mesoporous TiO2 was spin-coated on the substrate with a speed of 2500 rpm for 10s, and then 7000 rpm for 60s, from a diluted 50 nm particle paste (Dyesol) in a mixture of ethanol and terpineol. The weight ratio of TiO2 (Dyesol paste): Ethanol: terpineol was 1:2:4. After that, the substrate was immediately dried on a hotplate at 100°C for 2min, and then the substrates were sintered at 500°C overnight. The perovskite film was deposited by spin-coating onto the TiO2 substrate. The perovskite layer was deposited, in one-step, by spin coating the perovskite precursor solution which was prepared by mixing the methyl ammonium iodide (MAI), lead iodide (PbI2), lead chloride (PbCl2) in a mixed solvent of DMF and DMSO solution (volume ratio 9:1) with a ratio of 1:0.8:0.2 (Molar concentration = 0.9 M MAI : 0.72 M PbI2: 0.18 M PbCl2). The spin coating procedures consist of two stages: first 1000 rpm for 10 s, followed by 5000 rpm for 40 s. 500μL toluene was dropped on the spinning substrate during the second spin-coating step, 30s before the end of the procedure. The substrate was then annealed at 100°C for 5 min on a hotplate. Afterwards, the perovskite films were cooled down to room temperature and the HTMs were subsequently deposited on the top of the perovskite layer by spin coating at 4000 rpm for 30 s. The concentration of three HTMs were 16.2, 9.8 and 20.7 mg in 1ml of 1,1,2,2-tetrachloroethane for SGT-460, SGT-461 and SGT-462, respectively. The concentration of spiro-OMeTAD was 72.3 mg/1 mL chlorobenzene. When using the dopants, 17.5 μL of tris(bis(trifluoro-methyl-sulfonyl)imide) (Li-TFSI) stock solution (520 mg/1 mL in acetonitrile), 28.8 μL of tert-butylpyridine (t-BP) were added to HTMs solution as additives. Finally, 70 nm of gold was deposited using thermal evaporation.

Time-resolved photoluminescence decay Time-resolved photoluminescence (TR-PL) was measured by using a time-correlated single photon counting (TCSPC) spectrometer (SPC-130-EMN, Becker & Hickle inc.) equipped with a femtosecond light source (Home-built Cavity-dumped Ti :sapphire laser) and a single-photon Avalanche Photodiode (ID-100-50, IDQ inc.). The confocal setup using a parabolic mirror and a rod mirror was employed. Samples for TR-PL measurement, possessing the structure of pure glass/perovskite/HTMs, were excited by 30 fs, 390 nm laser pulses, which were incident on the HTM side (a shaking stage was used to avoid photo-damage), and the emission from the sample was collected at the wavelength of 780 nm.

Materials and synthesis All reactions were carried out under a nitrogen atmosphere. Solvents were distilled from appropriate reagents. Phosphorus(V) oxychloride, 2-bromothiophene, and malononitrile were purchased from Sigma Aldrich. 3-(Dicyanomethylidene) indan-1-one, 1,4-dibromo -2-nitrobenzene were purchased from Alfa Aesar. Oxindole, 3-ethylthiazolidine-2,4-dione were purchased from TCI. 3,8,13-Tribromo-5,10,15-trihexyl-10,15-dihydro-5*H*-diindolo[3,2-*a*:3',2'-*c*]carbazole (compound 3), [2] 6-bromo-4-hexyl-4*H*-thieno[3,2-*b*]indole (TI),[3] were synthesized by following the procedures.

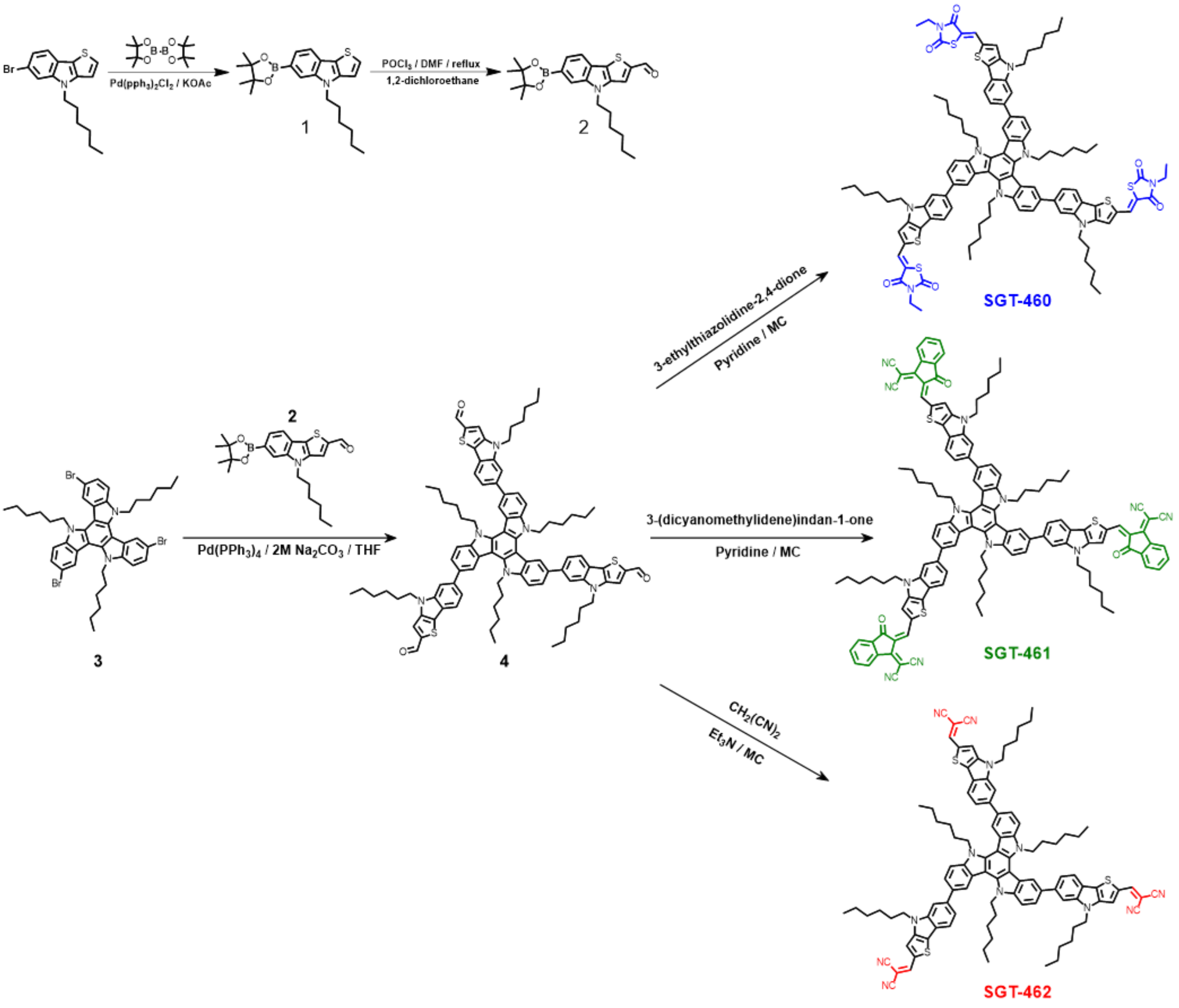

Scheme S1. Scheme of D-π-A structure HTMs synthesis.

4-hexyl-6-(4,4,5,5-tetramethyl-1,3,2-dioxaborolan-2-yl)-4*H*-thieno[3,2-*b*]indole (compound 1)

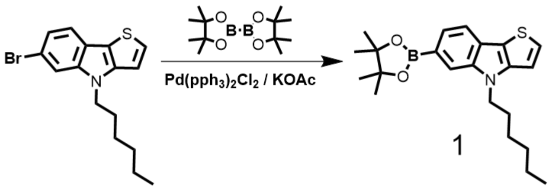

TI was added to an anhydrous solution of bis(pinacolato)diboron(1.2eq.), KOAc(4.5eq.), $PdCl_2(dppf)CH_2Cl_2$(5 mol%) in toluene (50 mL), then the mixture was refluxed at 110℃ for 5 h. After the reaction was completed, the reaction mixture was cooled to room temperature and the solvent was evaporated. Extraction was performed with CH_2Cl_2, and the organics layers were washed with H_2O and dried over anhydride sodium sulfate. Purification was done on a silica chromatography column using 10% $CHCl_3$ in hexane. The product isolated using the column chromatography was a yellow oil. (Yield 90%) ^{1}H NMR (300MHz, $CDCl_3$, ppm): δ 7.87 (s, 1H), δ 7.76-7.74 (d, 1H, J = 7.5Hz), δ 7.76-7.60 (d, 1H, J = 7.8Hz), δ 7.42-7.40 (d, 1H, J = 3.9Hz), δ 7.08-7.07 (d, 1H, J = 5.1Hz), δ 4.33-4.28 (t, 2H), δ 1.39-1.31 (m, 23H). HR-MS(MALDI-TOF): m/z. calcd, 383.2; found, 383.2129 (M^+).

4-hexyl-6-(4,4,5,5-tetramethyl-1,3,2-dioxaborolan-2-yl)-4*H*-thieno[3,2-*b*]indole-2-carbaldehyde (compound 2)

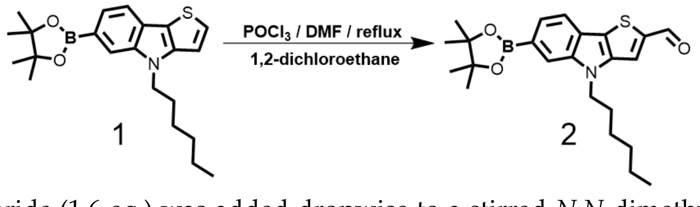

Phosphorous oxychloride (1.6 eq.) was added dropwise to a stirred *N*,*N*-dimethylformamide (1.6 eq.) at the temperature of ice water under an argon atmosphere. Then the mixture was added to the solution of compound 1 (1 eq.) in 1,2-dichloroethane (40 mL), dropwise at 0℃. After addition, the mixture was refluxed overnight. The resulting mixture was neutralized to pH = 7~8 with aqueous NaOH solution (20 wt%), extracted with CH_2Cl_2, and the residue was chromatographed on a silica gel column. The pure compound was obtained after column chromatography on silica gel using 10% ethyl acetate in hexane, gradually increasing the polarity up to 30% ethyl acetate in hexane. The yield was 1.5g (60%) as a yellow solid. ^1H NMR (300MHz, $CDCl_3$, ppm): δ 9.97(s, 1H), δ 7.89(s, 1H), δ 7.86-7.83(d, 1H, J = 8.1Hz), δ 7.73(s, 1H), δ7.67-7.64(d, 1H, J = 7.5Hz), δ 4.35-4.30 (t, 2H), δ 1.34-1.13 (m, 23H). HR-MS(MALDI-TOF): m/z. calcd, 411.2; found, 411.2028 (M^+).

6,6',6''-(5,10,15-trihexyl-10,15-dihydro-5*H*-diindolo[3,2-*a*:3',2'-c]carbazole-3,8,13-triyl)tris(4-hexyl-4*H*-thieno[3,2-*b*]indole-2-carbaldehyde) (compound 4)

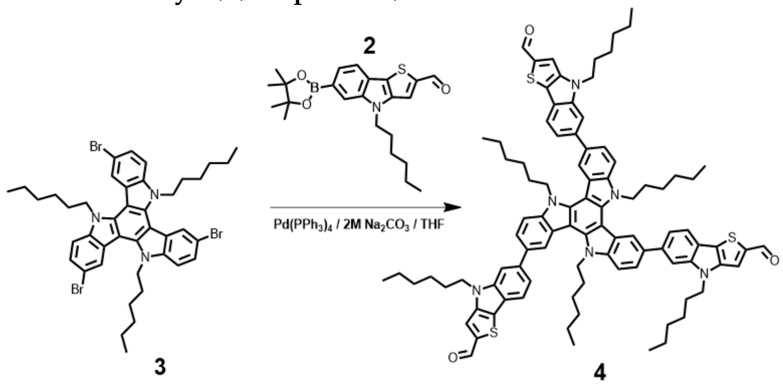

To a degassed mixture of compound 1 (1 eq.), compound 2 (5 eq.) in THF (30 ml) and 2M aqueous Na_2CO_3 (3 mL), $Pd(PPh_3)_4$ (20 mol%) was added under Ar_2. The resulting solution was refluxed to 80℃ overnight. After the reaction was completed, the reaction mixture was cooled to room temperature and the solvent was evaporated. The mixture was poured into water and extracted with CH_2Cl_2. The organic layer was dried over anhydrous sodium sulfate, filtered and evaporated to afford the crude compound. The crude product was purified by column chromatography on silica gel using the eluting solvent mixture of 30% acetone in hexane, to give pure compound as a yellow solid. The compound 3, isolated using the column chromatography, was a yellow solid. ^1H NMR (300MHz, $CDCl_3$, ppm): δ 9.99 (s, 3H), δ 8.42-8.40 (d, 3H, J = 8.4), δ 8.01-7.98 (d, 3H, J = 8.4), δ 7.93 (6H), δ 7.77 (s, 3H), δ 7.71-7.69 (6H), δ 5.07 (t, 18H), δ 4.41-4.39 (t, 18H), δ 2.10-0.76 (m, 66H). HR-MS(MALDI-TOF): m/z. calcd, 1447.7; found, 1447.7763 (M^+).

A general method for Knoevenagel condensation reaction:

Compound 4 (0.5g, 0.35 mmol, 1 eq.) and Acceptor [3-ethylthiazolidine-2,4-dione (0.23mL, 2.07 mmol, 6 eq.), 3-(dicyanomethylidene)indan-1-one (0.40g, 2.07mmol, 6eq.), and malononitrile (0.14g, 2.07mmol, 6 eq.)] were dissolved in dry CH_2Cl_2 (30 mL) and stirred for 1h with a few drops of triethylamine or pyridine at room temperature. After completion of the reaction monitored by TLC, the mixture was acidified with a few drops of conc. HCl solution. The reaction mixture was washed with water and the

organic layer extracted with CH_2Cl_2 dried over anhydrous sodium sulfate and filtered. Column chromatography on silica gel was performed to purify the final compounds.

(5Z,5'Z,5''Z)-5,5',5''-(((5,10,15-trihexyl-10,15-dihydro-5*H*-diindolo[3,2-*a*:3',2'-*c*] carbazole-3,8,13-triyl)tris(4-hexyl-4*H*-thieno[3,2-*b*]indole-6,2-diyl))tris(methanylylid ene))tris(3-ethylthiazolidine-2,4-dione) (SGT-460)

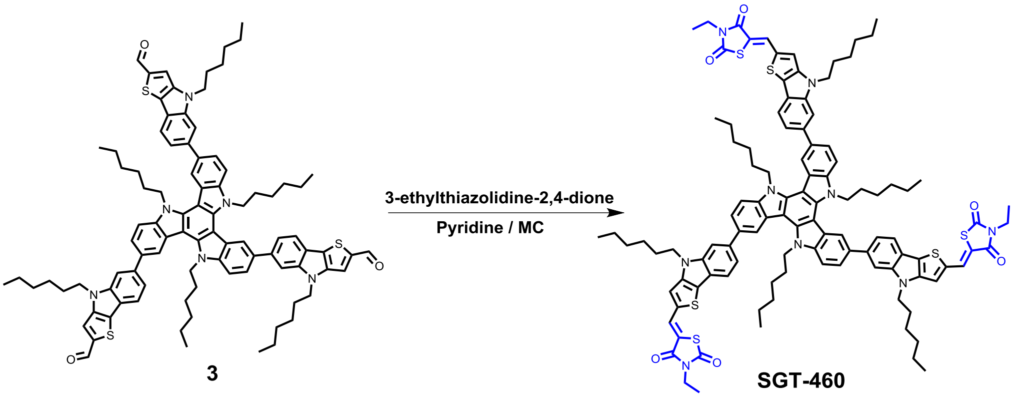

The pure compound was obtained after column chromatography on silica gel using eluent (THF : hexane = 2:1). gradually increasing the polarity up to eluent (THF : hexane = 3:1). ^{1}H NMR (300MHz, $CDCl_3$, ppm): δ 9.98 (s, 1H), δ 8.62 (s, 1H), δ7.77 (s, 1H), δ7.67-7.66 (d, 1H, J = 0.6), δ7.63-7.57 (d,1H, J = 3.6), δ 7.28 (s, 1H), δ 4.00-3.86 (m, 4H), δ 3.72-3.65 (m, 4H), δ 1.50-1.16 (m, 33H), δ 1.00-0.80 (m, 33H). HR-MS(MALDI-TOF): m/z. calcd, 1858.8; found, 1858.5592 (M^+).

2,2',2''-((2Z,2'Z,2''Z)-(((5,10,15-trihexyl-10,15-dihydro-5*H*-diindolo[3,2-*a*:3',2'-*c*] carbazole-3,8,13-triyl)tris(4-hexyl-4*H*-thieno[3,2-*b*]indole-6,2-diyl))tris(methanylylid ene))tris(3-oxo-2,3-dihydro-1*H*-indene-2,1-diylidene))trimalononitrile (SGT-461)

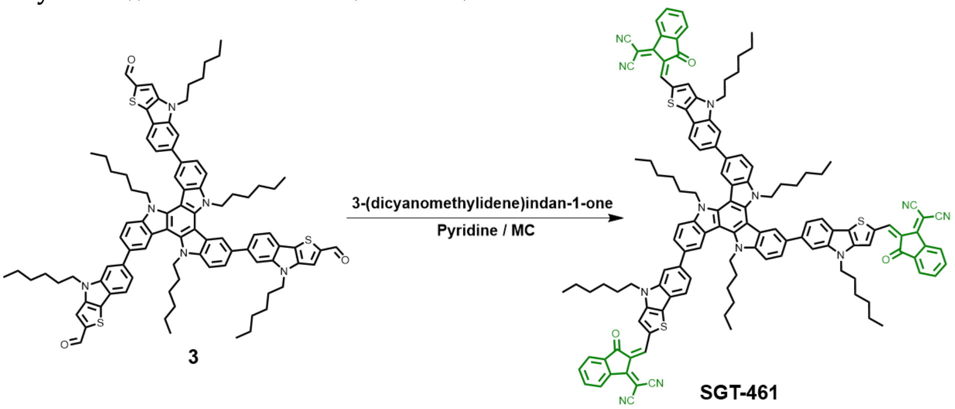

The pure compound was obtained after column chromatography on silica gel using eluent (THF : Hexane = 2:1). gradually increasing the polarity up to eluent (THF : Hexane = 3:1). ^{1}H NMR (300MHz, $CDCl_3$, ppm): δ 9.79 (s, 3H), δ 8.62-8.61 (d, 2H, J = 0.6) δ 8.12 (s, 1H), δ 7.75-7.57 (m, 3H), δ7.53-7.48 (d, 1H, J = 3), δ7.46-7.44 (d, 1H, J = 1.2), δ7.35 (s, 2H), δ 4.35-4.33 (6H), δ 4.25-4.28 (6H), δ 1.50-1.16 (m, 33H), δ 1.00-0.80 (m, 33H). HR-MS(MALDI-TOF): m/z. calcd, 1975.8; found, 1975.9447 (M^+).

2,2',2''-(((5,10,15-trihexyl-10,15-dihydro-5*H*-diindolo[3,2-*a*:3',2'-*c*]carbazole-3,8,13-triyl)tris(4-hexyl-4*H*-thieno[3,2-*b*]indole-6,2-diyl))tris(methanylylidene))trimalononitrile (SGT-462)

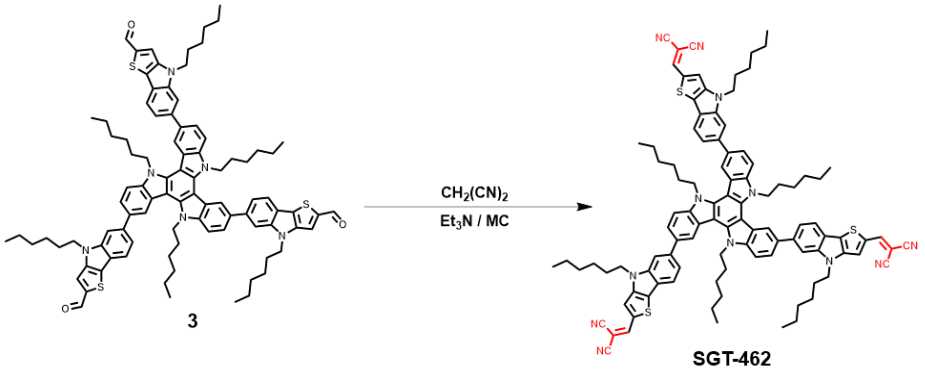

The pure compound was obtained after column chromatography on silica gel using eluent (THF : hexane = 3:1). ^{1}H NMR (300MHz, $(CD_3)_2CO$, ppm): δ 8.92 (s, 3H), δ 8.33 (s, 3H), δ 8.01 (s, 3H), δ 7.84-7.84 (d, 3H, J = 8.7), δ 7.77-7.73 (d, 3H, J = 2.4), δ 7.66-7.65 (d, 3H, J = 3.0), δ 6.99 (s, 3H), δ 6.88-6.84 (d, 3H, J = 6.9), δ 4.37-4.30 (6H), δ 4.27-4.24 (6H), δ 1.50-1.16 (m, 33H), δ 1.00-0.80 (m, 33H). HR-MS(MALDI-TOF): m/z. calcd, 1591.7; found, 1591.5931 (M^+).

NMR and Mass spectra

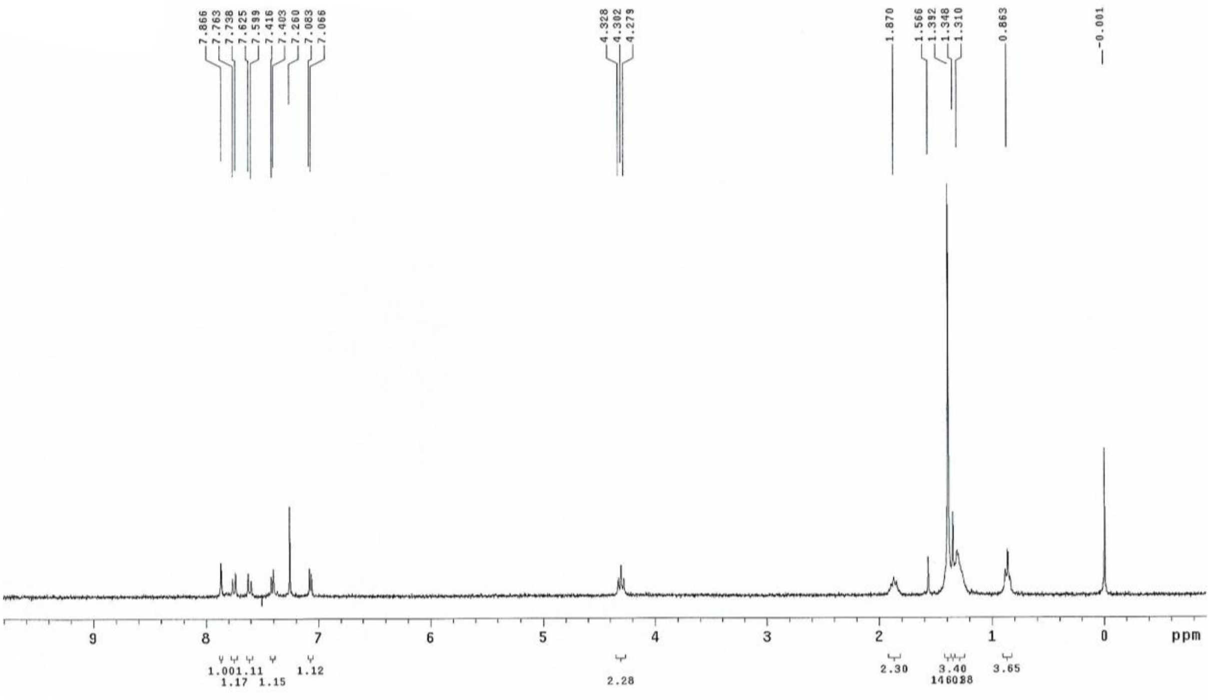

Figure S1. ^{1}H-NMR spectrum of 4-hexyl-6-(4,4,5,5-tetramethyl-1,3,2-dioxaborolan-2-yl)-4*H*-thieno[3,2-*b*]indole (compound 1)

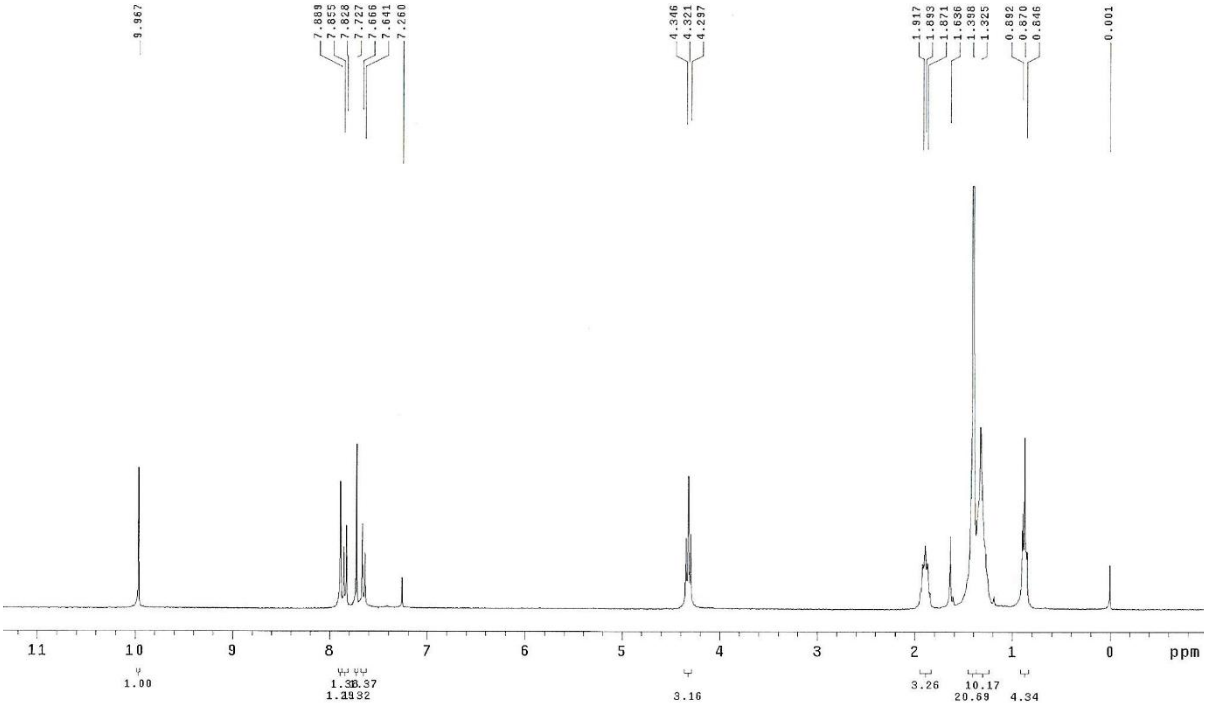

Figure S2. ^{1}H-NMR spectrum of 4-hexyl-6-(4,4,5,5-tetramethyl-1,3,2-dioxaborolan-2-yl)-4*H*-thieno[3,2-*b*]indole-2-carbaldehyde (compound 2)

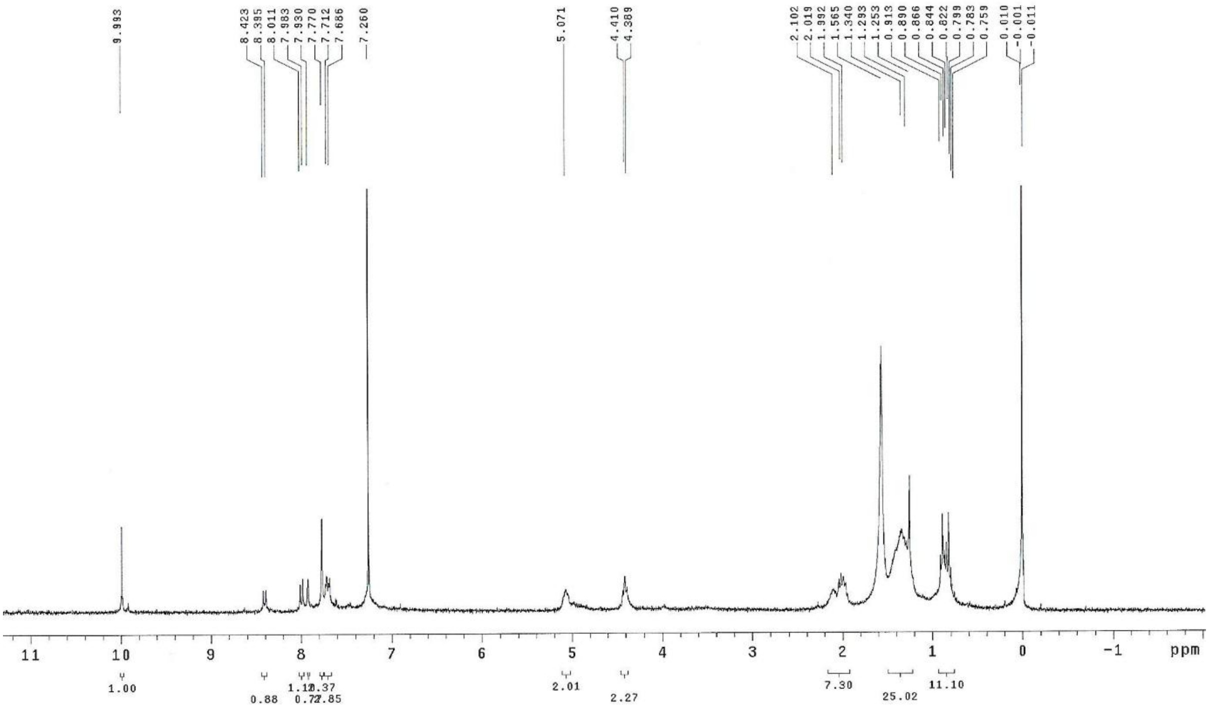

Figure S3. ^{1}H-NMR spectrum of 6,6',6''-(5,10,15-trihexyl-10,15-dihydro-5*H*-diindolo[3,2-*a*:3',2'-*c*]carbazole-3,8,13-triyl)tris(4-hexyl-4*H*-thieno[3,2-*b*]indole-2-carbaldehyde) (compound 4)

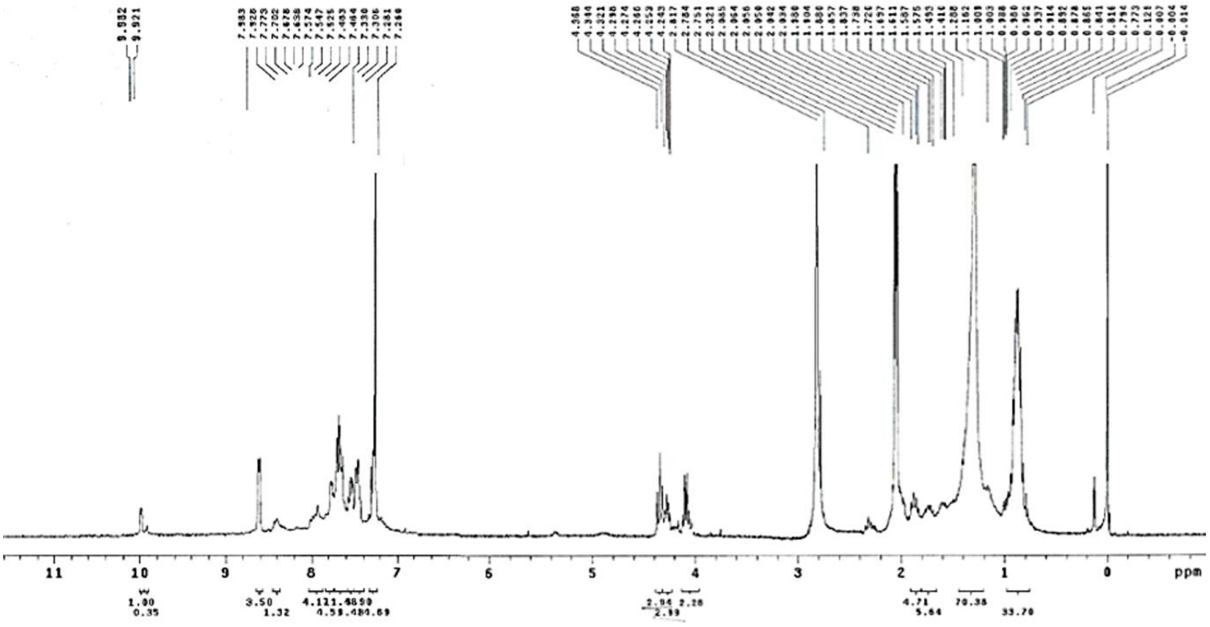

Figure S4. ^{1}H-NMR spectrum of (5Z,5'Z,5''Z)-5,5',5''-(((5,10,15-trihexyl-10,15-dihydro-5*H*-diindolo[3,2-*a*:3',2'-*c*]carbazole-3,8,13-triyl)tris(4-hexyl-4*H*-thieno[3,2-*b*]indole-6,2-diyl))tris (methanylylidene))tris(3-ethylthiazolidine-2,4-dione) (**SGT-460**)

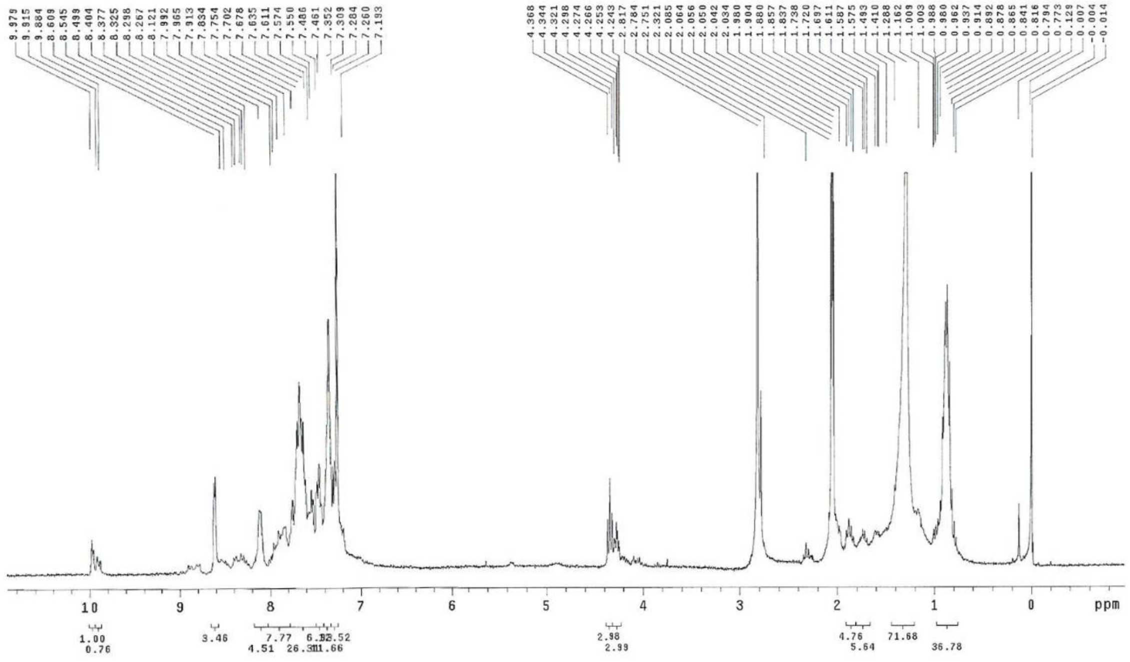

Figure S5. ^{1}H-NMR spectrum of 2,2',2''-((2Z,2'Z,2''Z)-(((5,10,15-trihexyl-10,15-dihydro-5*H*-diindolo[3,2-*a*:3',2'-*c*]carbazole-3,8,13-triyl)tris(4-hexyl-4*H*-thieno[3,2-*b*]indole-6,2-diyl))tris (methanylylidene))tris(3-oxo-2,3-dihydro-1*H*-indene-2,1-diylidene))trimalononitrile (**SGT-461**)

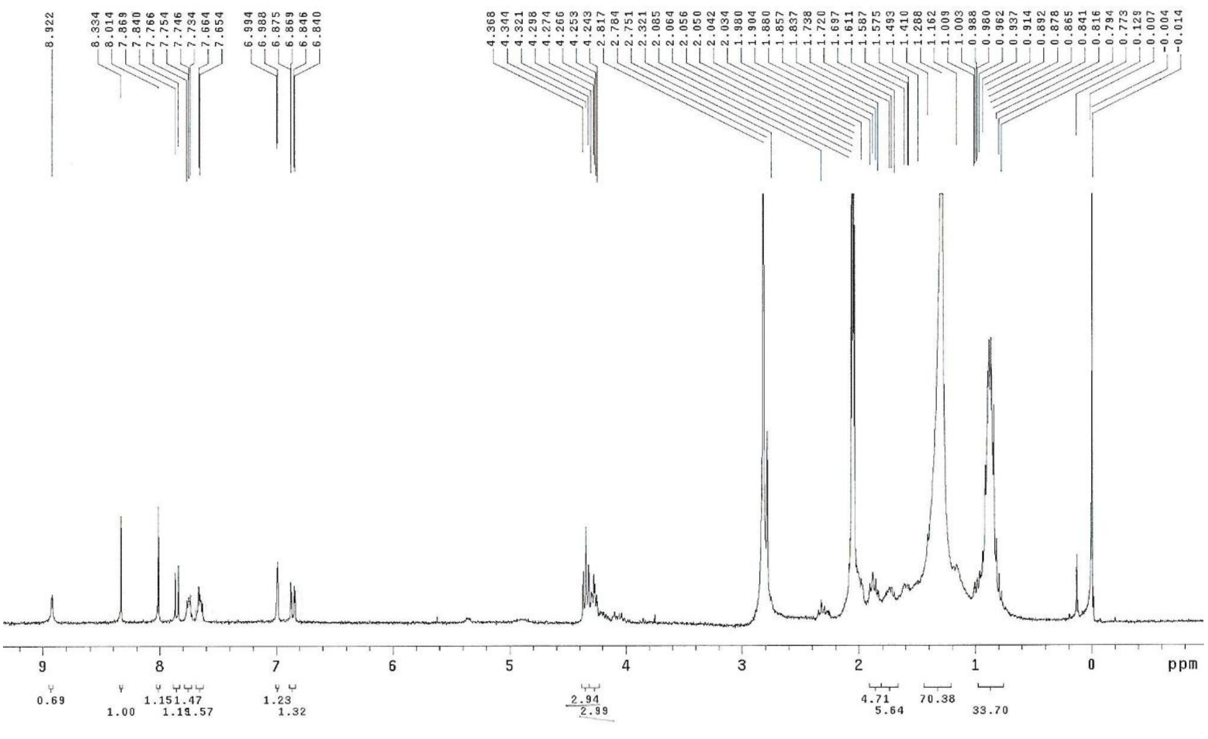

Figure S6. ^{1}H-NMR spectrum of 2,2',2''-(((5,10,15-trihexyl-10,15-dihydro-5*H*-diindolo[3,2-*a*:3',2'-*c*]carbazole-3,8,13-triyl)tris(4-hexyl-4*H*-thieno[3,2-*b*]indole-6,2-diyl))tris(methanylylidene))trimalononitrile (**SGT-462**)

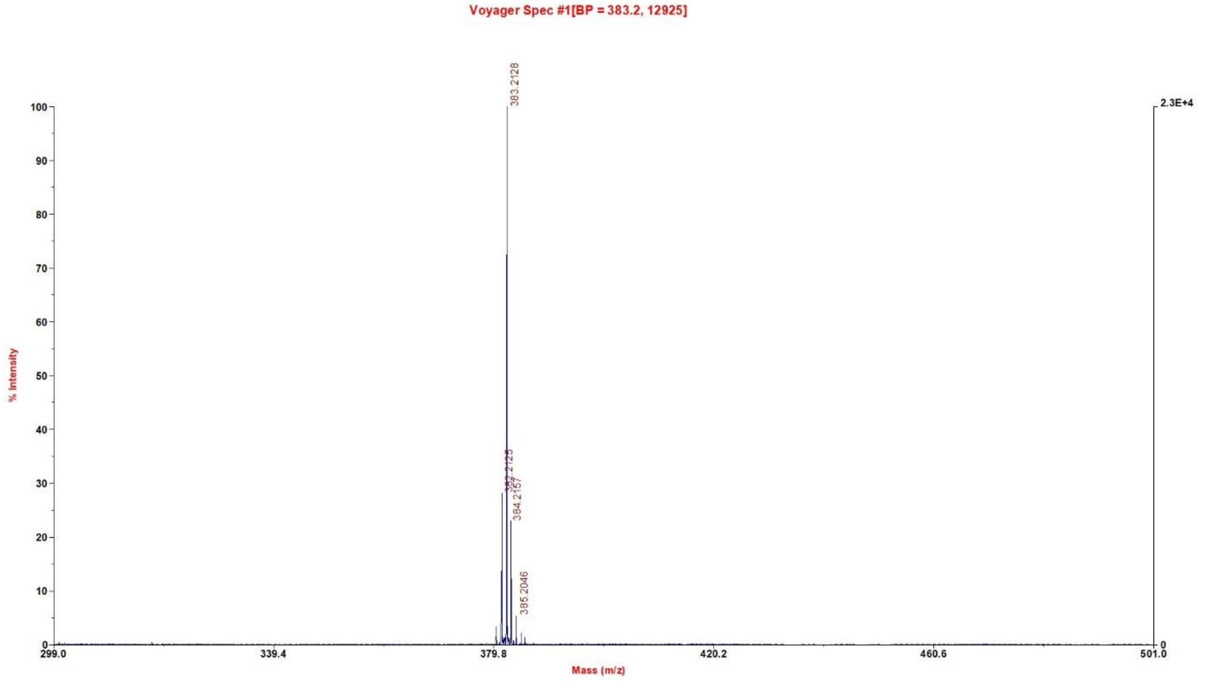

Figure S7. Matrix-Assisted Laser Desorption Ionization-Time-of-Flight (MALDI-TOF) spectrum of 4-hexyl-6-(4,4,5,5-tetramethyl-1,3,2-dioxaborolan-2-yl)-4*H*-thieno[3,2-*b*]indole (compound 1)

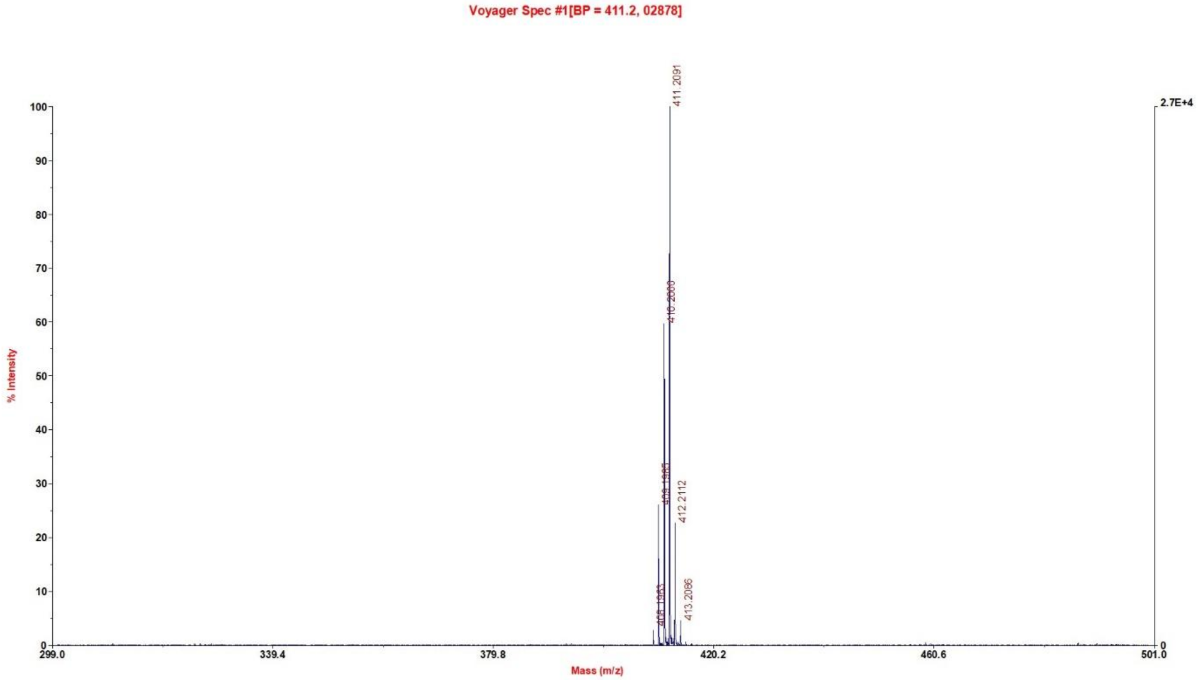

Figure S8. MALDI-TOF spectrum of 4-hexyl-6-(4,4,5,5-tetramethyl-1,3,2-dioxaborolan-2-yl)-4*H*-thieno[3,2-*b*]indole-2-carbaldehyde (compound 2)

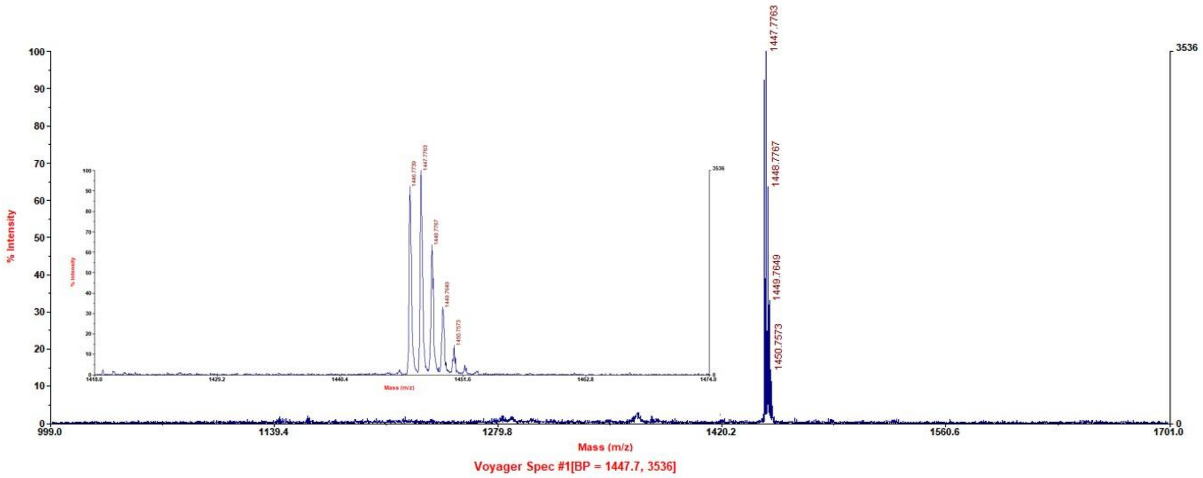

Figure S9. MALDI-TOF spectrum of 6,6',6''-(5,10,15-trihexyl-10,15-dihydro-5*H*-diindolo[3,2-*a*:3',2'-*c*]carbazole-3,8,13-triyl)tris(4-hexyl-4*H*-thieno[3,2-*b*]indole-2-carbaldehyde) (compound 4)

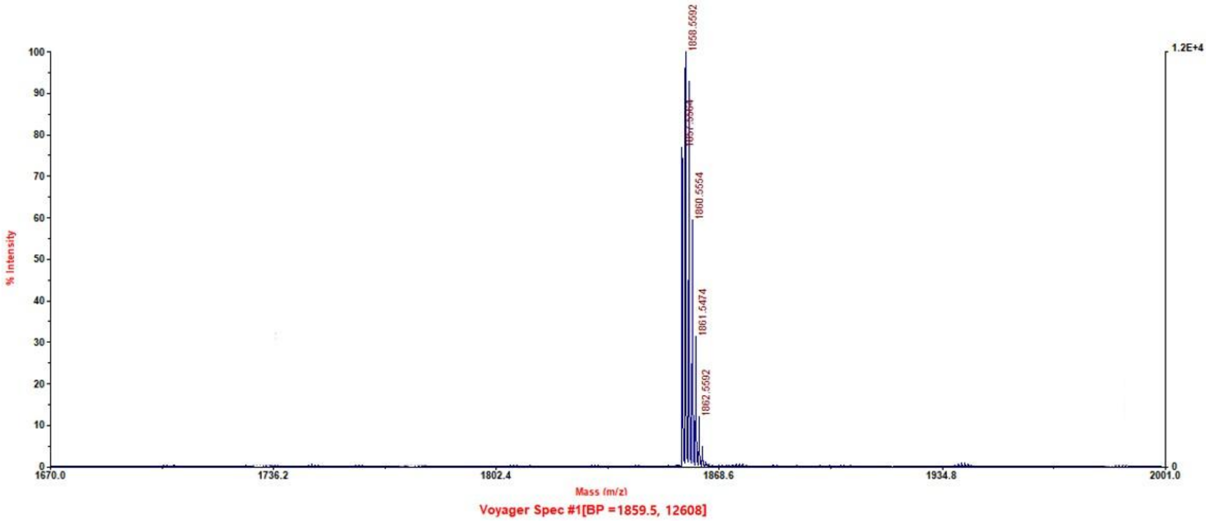

Figure S10. MALDI-TOF spectrum of (5Z,5'Z,5''Z)-5,5',5''-(((5,10,15-trihexyl-10,15-dihydro-5*H*-diindolo[3,2-*a*:3',2'-*c*]carbazole-3,8,13-triyl)tris(4-hexyl-4*H*-thieno[3,2-*b*]indole-6,2-diyl))tris(methanylylidene))tris(3-ethylthiazolidine-2,4-dione) (**SGT-460**)

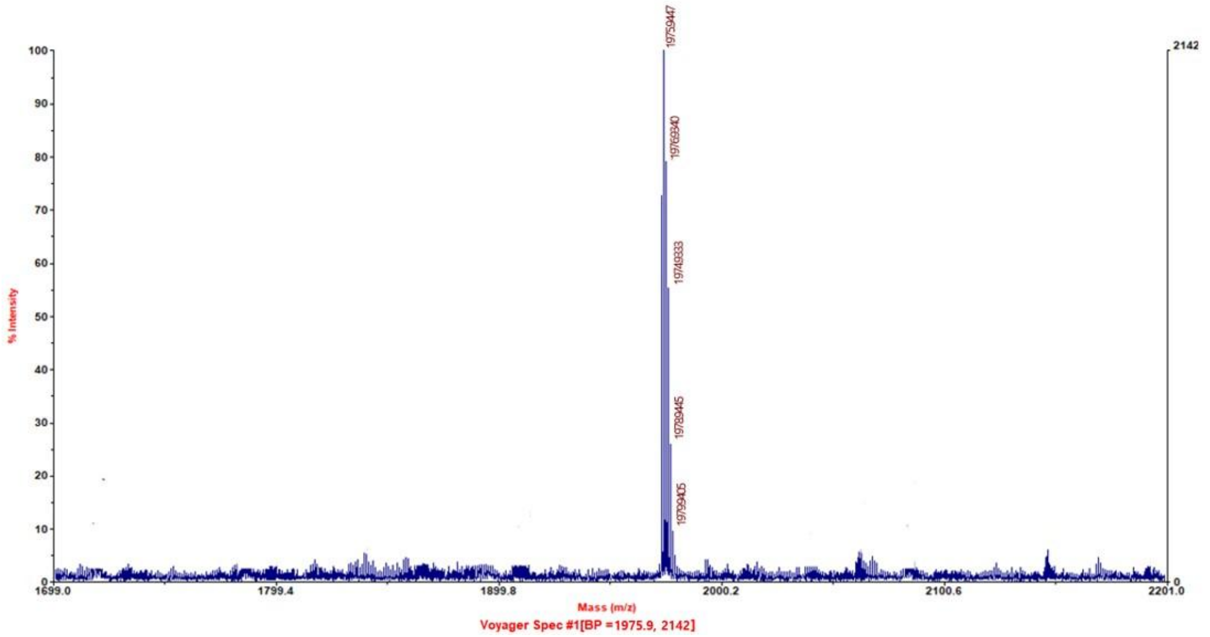

Figure S11. MALDI-TOF spectrum of 2,2',2''-((2Z,2'Z,2''Z)-(((5,10,15-trihexyl-10,15-dihydro-5*H*-diindolo[3,2-*a*:3',2'-*c*]carbazole-3,8,13-triyl)tris(4-hexyl-4*H*-thieno[3,2-*b*]indole-6,2-diyl))tris(methanylylidene))tris(3-oxo-2,3-dihydro-1*H*-indene-2,1-diylidene))trimalononitrile (**SGT-461**)

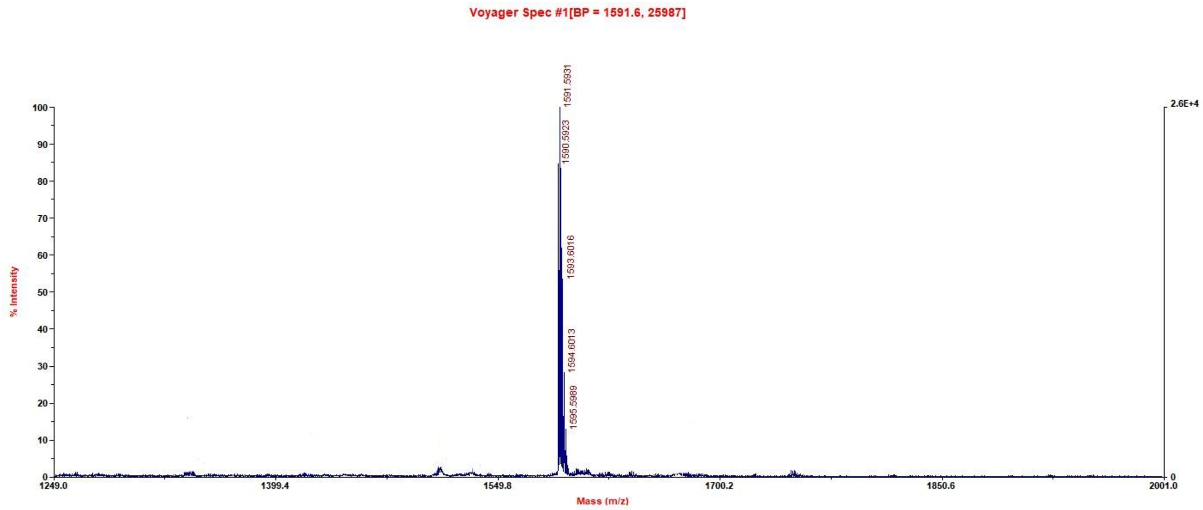

Figure S12. MALDI-TOF spectrum of 2,2',2''-(((5,10,15-trihexyl-10,15-dihydro-5*H*-diindolo[3,2-*a*:3',2'-*c*]carbazole-3,8,13-triyl)tris(4-hexyl-4*H*-thieno[3,2-*b*]indole-6,2-diyl))tris(methanylylidene))trimalononitrile (**SGT-462**)

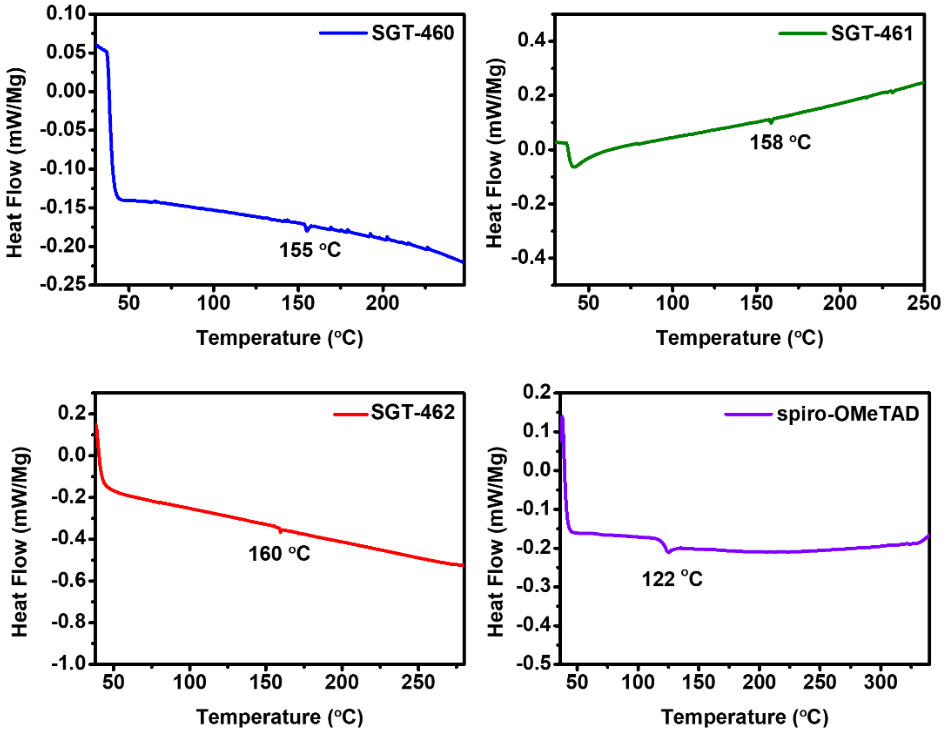

Figure S13. Differential scanning calorimetry (DSC) traces of HTMs.

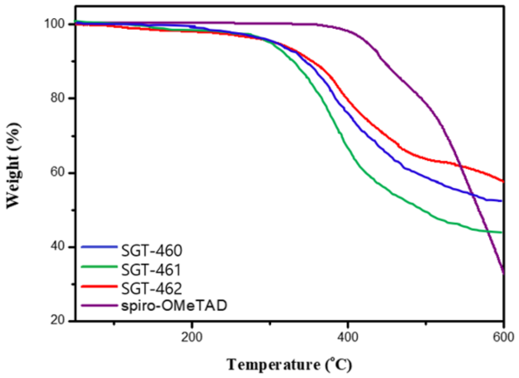

Figure S14. Thermogravimetric analysis (TGA) traces of HTMs

Table S1. Thermal properties of the synthesized HTMs and spiro-OMeTAD.

HTM	T_g [1] (°C)	T_d [2] (°C)
**SGT-460**	155	345
**SGT-461**	158	339
**SGT-462**	160	357
spiro-OMeTAD	122	406

[1] Glass transition (collected from the second circle of the DSC curve). [2] Degradation temperature observed from TGA measurements (10 _C min_1 under a N2 atmosphere).

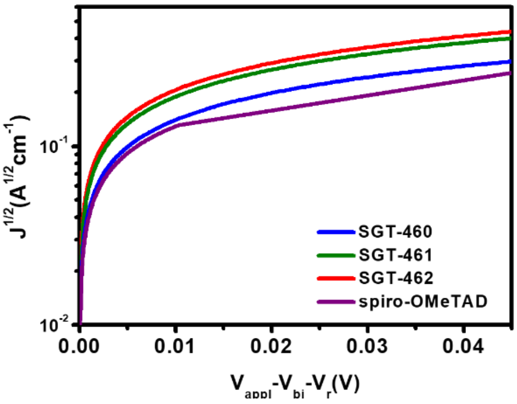

Figure S15. *J–V* characteristics of **SGT-460**, **SGT-461**, **SGT-462** and spiro-OMeTAD (w/o dopants) in hole-only devices.

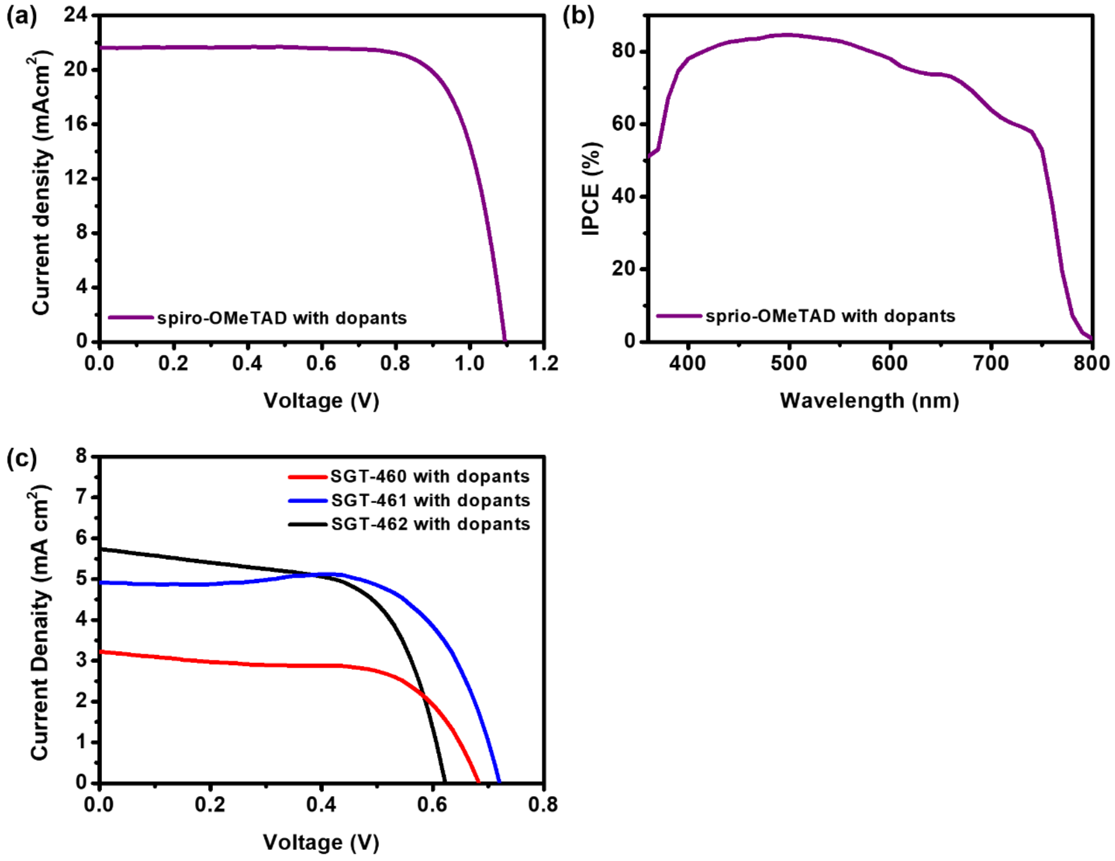

Figure S16. *J-V* curve of the PrSC based on (a) spiro-OMeTAD with dopants and (b) the corresponding IPCE spectrum. (c) *J-V* curves of the PrSCs based on **SGT-460**, **SGT-461** and **SGT-462** with dopants

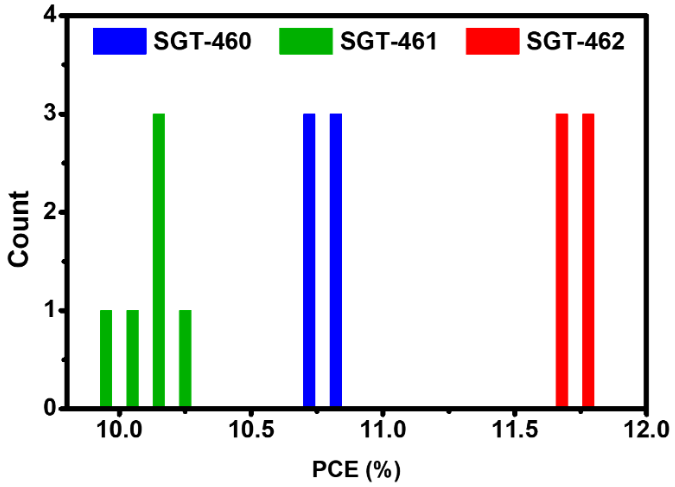

Figure S17. Histograms for three dopant-free HTMs based PrSCs.

Table S2. Photovoltaic performances of the PrSCs based on spiro-OMeTAD and three SGT-HTMs with dopants

HTM	J_{SC} (mA cm^{-2})	V_{OC} (V)	*FF* (%)	PCE (%)[1]
spiro-OMeTAD with dopants	21.62	1.094	75.5	17.8
SGT-460 with dopants	3.56	0.622	62.1	1.38
SGT-461 with dopants	2.00	0.683	62.9	0.86
SGT-462 with dopants	3.06	0.720	69.8	1.54

[1] measured under simulated AM 1.5 G irradiation.

Table S3. The solubility parameters of three dopant-free HTMs in organic solvents[a].

HTM	Chlorobenzene (mg ml^{-1})	1,2-dichlorobenzene (mg ml^{-1})	1,1,2,2-tetrachloroethane (mg ml^{-1})
**SGT-460**	4.5	4.2	16.2
**SGT-461**	2.4	2.1	9.8
**SGT-462**	6.8	7.1	20.7

Table S4. The fitting results of time resolved photoluminescence (TRPL) profiles

HTM	A_1 (%)	τ_1 (ns)	A_2 (%)	τ_2 (ns)	R1[1]	R2[1]	$\tau_{interface}$[2]	τ_{ct}[3]	CTE(%)[4]
bare perovskite	69.4	23.4	30.6	107.8	0.69	0.31	49.2		
**SGT-460**	70.2	10.4	29.8	50.4	0.70	0.30	16.4	24.6	66.7
**SGT-461**	78.9	11.8	21.1	50.4	0.79	0.21	19.9	33.5	59.5
**SGT-462**	83.5	10.4	16.5	36.8	0.84	0.17	15.2	21.9	69.2
spiro-OMeTAD	87.7	6.2	42.2	21.8	0.68	0.32	11.3	14.6	77.1

[1] R_1 and R_2 are relative ratio factors, given by $R_1 = \frac{A_1}{A_1+A_2}, R_2 = \frac{A_2}{A_1+A_2}$.

[2] The PL lifetime of each pyrex glass/TiO_2/$CH_3NH_3PbI_{3-x}Cl_x$/HTM sample corresponds to the amplitude weighted average lifetime of a biexponential decay fit.
(Time constant for the TR-PL decay of the neat $CH_3NH_3PbI_{3-x}Cl_x$ film)

[3] τ_{CT} was calculated by using the relationship of $\frac{1}{\tau_{interface}} = \frac{1}{\tau_{perovskite}} + \frac{1}{\tau_{CT}}$.

[4] Charge-transfer efficiency (CTE) was calculated by using equation: $CTE = \frac{k_{CT}}{k_{interface}} = \frac{\tau_{interface}}{\tau_{CT}}$.

Note : the fitting functions of the biexponential decay equation. $I(t) = I_0 + A_1 e^{\frac{-t}{\tau_1}} + A_2 e^{\frac{-t}{\tau_2}}$.

Supplementary References

[1] C. Lu, M. Paramasivam, K. Park, C. H. Kim, H. K. Kim, Phenothiazine functionalized multifunctional A–π–D–π–D–π–A-type hole-transporting materials via sequential C–H arylation approach for efficient and stable perovskite solar cells. *ACS Appl. Mater. Interfaces* **2019**, 11, 14011-14022, doi.org/10.1021/acsami.8b20646.

[2] C. Huang, W. Fu, C-Z. Li, Z. Zhang, W. Qiu, M. Shi, Dopant-free hole-transporting material with a C3h symmetrical truxene core for highly efficient perovskite solar cells. *J. Am. Chem. Soc.* **2016**, 138,

2528-2531, doi.org/10.1021/jacs.6b00039.
[3] Y. K. Eom, S. H. Kang, I. T. Choi, Y. Yoo, J. Kim, H. K. Kim, Significant light absorption enhancement by a single heterocyclic unit change in the π-bridge moiety from thieno[3,2-*b*]benzothiophene to thieno[3,2-*b*]indole for high performance dye-sensitized and tandem solar cells. J. Mater. Chem. A **2017**, 5, 2297-2308, doi.org/10.1039/C6TA09836C.
